# Current Challenges in Studying Alternative Splicing in Plants: The Case of *Physcomitrella patens* SR Proteins

**DOI:** 10.3389/fpls.2020.00286

**Published:** 2020-03-24

**Authors:** José Pedro Melo, Maria Kalyna, Paula Duque

**Affiliations:** ^1^Instituto Gulbenkian de Ciência (IGC), Oeiras, Portugal; ^2^Department of Applied Genetics and Cell Biology, BOKU – University of Natural Resources and Life Sciences, Vienna, Austria

**Keywords:** *Physcomitrella patens*, *Arabidopsis thaliana*, RNA splicing, alternative splicing, SR proteins, gene annotation, stress, evolution

## Abstract

To colonize different terrestrial habitats, early land plants had to overcome the challenge of coping with harsh new environments. Alternative splicing – an RNA processing mechanism through which splice sites are differentially recognized, originating multiple transcripts and potentially different proteins from the same gene – can be key for plant stress tolerance. Serine/arginine-rich (SR) proteins constitute an evolutionarily conserved family of major alternative splicing regulators that in plants subdivides into six subfamilies. Despite being well studied in animals and a few plant species, such as the model angiosperm *Arabidopsis thaliana* and the crop *Oryza sativa*, little is known of these splicing factors in early land plants. Establishing the whole complement of SR proteins in different species is essential to understand the functional and evolutionary significance of alternative splicing. An *in silico* search for SR proteins in the extant moss *Physcomitrella patens* revealed inconsistencies both in the published data and available databases, likely arising from automatic annotation lacking adequate manual curation. These misannotations interfere with the description not only of the number and subfamily classification of Physcomitrella SR proteins but also of their domain architecture, potentially hindering the elucidation of their molecular functions. We therefore advise caution when looking into *P. patens* genomic resources. Our systematic survey nonetheless confidently identified 16 *P. patens* SR proteins that fall into the six described subfamilies and represent counterparts of well-established members in *Arabidopsis* and rice. Intensified research efforts should disclose whether SR proteins were already determining alternative splicing modulation and stress tolerance in early land plants.

## The Adaptation of Plants to Life on Land

About 500 million years ago the first plants colonized land, resulting in one of the most important events in the history of life on Earth (Morris et al., 2018). The evolution and diversification of land plants shaped the biosphere and created the conditions that allowed the colonization of land by metazoans and the subsequent formation of complex terrestrial habitats (Floyd and Bowman, 2007).

However, terrestrial and aquatic environments differ greatly, mainly regarding water, temperature and radiation exposure. Thus, plants had to develop a number of regulatory cellular and physiological traits to cope with these adverse conditions (Kenrick and Crane, 1997). Some of these features are already present in charophycean algae, such as a three-dimensional, predominant haploid gametophyte, but others evolved during or after colonization. Furthermore, some appeared *de novo* while others evolved from existing traits. Some appeared only once, others originated multiple times, whereas others were gained and then lost in some lineages. Key innovations include the internalization of vital functions and organs and the development of impermeable exterior surfaces, leading to the appearance of specialized sexual organs (gametangia), vascular tissues, stomata, symbiosis with fungi, branched shoots, leaves, roots, seeds and flowers (Kenrick and Crane, 1997; Heckman et al., 2001; Floyd and Bowman, 2007; Rensing et al., 2008; Morris et al., 2018; One Thousand Plant Transcriptomes Initiative, 2019). At the cellular level, plants evolved better osmoregulation, desiccation, freezing and heat resistance as well as enhanced DNA repair mechanisms (Gensel, 2008; Ju et al., 2015; de Vries and Archibald, 2018).

Most of these traits evolved prior to the appearance of flowering plants, highlighting the importance of establishing and studying model species that represent all major plant groups. The extant moss *Physcomitrella patens* occupies a key position in the evolution of plants, between aquatic green algae and vascular plants, and presents characteristics of both, rendering it an invaluable tool to study the onset of terrestrial-based plant life, as well as the appearance and diversification of important traits for modern day crops (Rensing et al., 2008; Smidkova et al., 2010).

## Alternative Splicing as a Means of Adapting to a Changing Environment

A characteristic of eukaryotic protein-coding genes is the presence of segments of non-coding DNA, called introns, interspaced with coding DNA segments, the exons. When the precursor mRNA (pre-mRNA) is processed, specific splice sites are recognized, the introns are removed and the exons joined together (or spliced). However, splice sites can be differentially recognized, leading to the inclusion or removal of different segments of RNA, resulting in multiple transcripts, and potentially proteins, originating from the same gene. This mechanism is termed alternative splicing and represents an effective means of both increasing transcriptome and proteome diversity and regulating gene expression by affecting the stability of the transcripts (Shang et al., 2017). Alternative splicing can often generate transcripts with premature termination codons, which are targeted to degradation by nonsense-mediated mRNA decay (NMD). Coupling of NMD with alternative splicing can represent an important means of regulating the abundance of certain proteins, such as splicing regulators, in a homeostatic feedback loop (Nasif et al., 2018).

A large proportion of genes is known to be alternatively spliced, with recent studies indicating that up to 70% of plant multi-exon genes undergo alternative splicing (Marquez et al., 2012; Chamala et al., 2015; Zhang et al., 2017). This number has been increasing over time as genome annotations improve and the use of next generation sequencing provides not only more but also deeper data (Syed et al., 2012). Estimates of alternative splicing rates in *P. patens* show a similar percentage (58%) of genes being alternatively spliced (Zimmer et al., 2013; Lang et al., 2016), although the actual number may be higher.

Alternative splicing is involved in numerous biological processes. In plants, it is especially important in the response to external cues, particularly environmental stresses (Staiger and Brown, 2013), and is hence likely to have played an important role in the process of land colonization (Mastrangelo et al., 2012). However, this hypothesis remains to be adequately tested. In *P. patens*, alternative splicing has been shown to be regulated by light (Wu et al., 2014) and to improve tolerance to heat stress (Chang et al., 2014). These findings are consistent with this posttranscriptional mechanism having helped early land plants cope with adverse terrestrial conditions. On the other hand, though further studies are needed to support this notion, it has also been reported that alternative splicing does not significantly affect proteome diversity in *P. patens* (Fesenko et al., 2017), suggesting that this mechanism could increase stress tolerance through the modulation of transcript stability and thereby protein abundance. It has also been shown that about 32% of alternatively spliced genes in *P. patens* are targeted to NMD, pointing to an important role of this mRNA degradation mechanism in gene regulation (Lloyd et al., 2018). The scarcity of available data and the existence of conflicting evidence underscore the need for thorough bioinformatics and evolutionary studies to address the role of alternative splicing in plant conquest of land.

## SR Proteins: a Highly Conserved Family of Key Alternative Splicing Regulators

Serine/arginine-rich (SR) proteins represent an important family of RNA-binding proteins that is highly conserved among eukaryotes. Their most well-known role is in the regulation of pre-mRNA splicing, being involved in the recognition of splice sites and recruitment and assembly of the spliceosome. Most importantly, these proteins influence the recognition of splice sites by core spliceosomal components and are thus major modulators of alternative splicing. Nonetheless, these are not the only known functions of SR proteins. Several studies in both animal and plant systems have also unveiled a myriad of other functions, such as genome maintenance, mRNA stability and export, and oncogenic transformation (Huang and Steitz, 2005; Tillemans et al., 2006; Long and Caceres, 2009; Zhong et al., 2009; Twyffels et al., 2011).

Their multifunctional roles and the historical timing of their discovery has led to several distinct SR protein classifications, an issue that was addressed almost a decade ago by Manley and Krainer (2010), who proposed a standardization of the definition and nomenclature of SR proteins. This was mostly based on mammalian proteins, and adapting the system to plants proved to be an arduous task due to the higher number and diversity of family members. In addition, some plant-specific SR proteins do not present orthologs in mammals and exhibit unique features that did not fall under Manley and Krainer’s definition. For this reason, in the same year, Barta et al. (2010) proposed an updated nomenclature for plant SR proteins to facilitate the assignment and comparison of these proteins across plant species.

**FIGURE 1:**
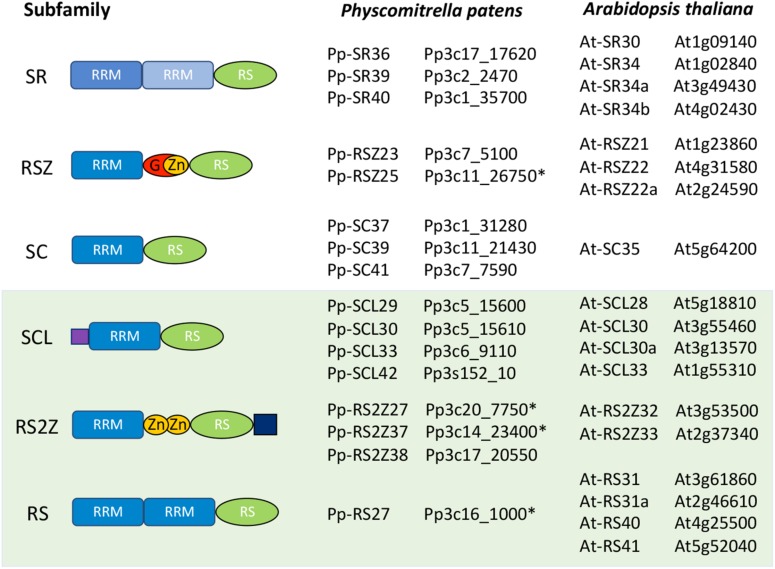
Schematic representation of the *Arabidopsis thaliana* and *Physcomitrella patens* SR protein families. All SR proteins are characterized by at least one RNA Recognition Motif (RRM) and an arginine/serine-rich (RS) region. Proteins in the RSZ subfamily include a glycine-rich region (G) after the first RRM. RSZ and RS2Z proteins also contain one and two zinc knuckles (Zn), respectively, with RS2Z members harboring an SP-rich extension after the RS region. The SCL subfamily is characterized by an N-terminal charged extension. The light-green shaded box indicates the three plant-specific SR protein subfamilies. Genes with incorrect reference models in the publicly available databases are marked with an asterisk (^∗^).

According to the established definition for plants, SR proteins are characterized by the presence of one or two N-terminal RNA Recognition Motifs (RRMs) and a C-terminal arginine/serine-rich (RS) region of at least 50 amino acids with a minimum of 20% RS or SR dipeptides (Barta et al., 2010 and Figure 1). Based on this definition, the genomes of the dicotyledonous model plant species *Arabidopsis thaliana* and the monocot crop *Oryza sativa* (rice) encode 18 and 22 SR proteins, respectively, which are classified into six subfamilies according to their protein domain architecture. Three of these subfamilies have orthologs in mammals - the SR subfamily is orthologous to the mammalian SRSF1/SF2/ASF, the RSZ subfamily to SRSF7/9G8 and the SC subfamily to SRSF2/SC35. The remaining three subfamilies, however, are plant-specific, presenting no clear orthologs in animals. There are also a few proteins that contain an RRM and an RS region, but are nevertheless no longer considered SR proteins due to the presence of an additional N-terminal RS region and are therefore named SR-like.

One peculiarity of plant SR genes is that many occur as duplicated pairs of paralogs, thus explaining why there are almost twice as many as in humans (Kalyna and Barta, 2004). Genome duplications, either whole-genome or large segmental duplications, are very common throughout the evolution of several plant species and lineages. However, most of what is known has been studied in angiosperms, such as Arabidopsis and rice, with only occasional reports providing insight into earlier plants (Adams and Wendel, 2005; Soltis et al., 2015; Panchy et al., 2016; Clark and Donoghue, 2018; Wu et al., 2020). Interestingly, extant mosses such as *P. patens*, but not hornworts and liverworts, also underwent genome duplications. More specifically, *P. patens* appears to have undergone two whole-genome duplication events, one 40–48 million years ago (Mya) and the other 27–35 Mya, giving rise to the current 27 chromosomes and ∼33,000 genes, a similar number to *A. thaliana* (Rensing et al., 2007, 2013; Lang et al., 2018). Another remarkable feature of plant SR genes is that they undergo highly conserved alternative splicing events in their longest introns, which in some cases has been maintained from *P. patens* or the single cell green alga *Chlamydomonas reinhardtii* to dicots throughout ∼1.1 billion years of evolution (Iida and Go, 2006; Kalyna et al., 2006). Interestingly, alternative splice sites in SR genes of the RS and RS2Z subfamilies are embedded in ultraconserved regions preserved for >400 million years of land plant evolution (Kalyna et al., 2006). A similar feature has also been observed for mouse and human SR genes (Lareau et al., 2007), pointing to the importance of this mode of regulation. Establishing the whole complement of SR proteins in *P. patens* would prove invaluable to understand both the general regulation of these RNA-binding proteins and their impact on alternative splicing in an early land plant.

## How Many SR Proteins Does the *Physcomitrella Patens* Genome Encode?

A few individual *P. patens* SR proteins have been previously reported and studied by different research groups (Iida and Go, 2006; Kalyna et al., 2006; Richardson et al., 2011; Califice et al., 2012; Rauch et al., 2014; Fesenko et al., 2017; Lloyd et al., 2018), but the gene family has hitherto not been addressed as a whole. Furthermore, not all studies report the same complement, subfamily classification or coding sequences, underscoring the need for revisiting these analyses. We conducted a comprehensive *in silico* search for SR genes in *P. patens* to determine their number and whether they fall under the current definition of this family as well as to establish reference protein sequences and domain architectures for each Physcomitrella SR protein.

A BLASTp search against the *P. patens* proteome using each *A. thaliana* SR protein sequence in the NCBI^1^, CoGe^2^ and Plaza^3^ databases yielded a list of 18 candidate *P. patens* SR proteins. However, after analyzing the sequences and metadata, including intron/exon positions, genome location, syntheny and expression data, in JBrowse, manual curation of the retrieved sequences resulted in the identification of only 16 *bona fide P. patens* SR proteins (Figure 1).

## Inconsistencies Within the Available Databases

Our initial BLAST search identified three *P. patens* proteins belonging to the RSZ subfamily. However, despite being annotated as two different genes, Pp3c11_26740V3.1 and Pp3c11_26750V3.1 occupy the same position in the genome (Supplementary Figure S1). We considered Pp3c11_26750V3.1 as the correct gene, as it is the longest annotation of the two and is unequivocally supported by EST, cDNA and RNA-seq data.

Another identified inconsistency related to Pp3c1_31300V3.1 from the SC subfamily. This gene includes a very short open reading frame (ORF) that does not support a full-length protein and is preceded by an unusually long 5′ UTR (Supplementary Figure S2). It is positioned next to another member of the SC subfamily, Pp3c1_31280V3.1, with which it shares a similar sequence.

Noteworthily, some of the retrieved genes do not present annotated untranslated regions (UTRs), and the annotated exon and intron positions are not supported by the EST, cDNA, and RNA-seq data available in NCBI, CoGe or Phytozome^4^. One such example is the Pp3c16_1000V3.1 gene model, which does not include an annotated 5′UTR, and whose annotated first exon is not supported by experimental evidence. Moreover, although the second exon is supported by EST, cDNA and RNA-seq data, its sequence harbors no AUG. The first AUG is found only in the third exon. As such, the correct protein for this member of the RS subfamily appears to start in the third annotated exon (Supplementary Figure S3), with the second exon likely being included in the 5′UTR. This is likely due to an automatic annotation of the genome lacking proper manual curation. Another such example is Pp3c20_7750V3.1 from the RS2Z subfamily, whose reference gene model also lacks annotated UTRs, despite the fact that both alternative transcripts have annotated UTRs (Supplementary Figure S4).

An additional misannotation occurs in Pp3c14_23400V3.1, another RS2Z subfamily member, for which the first exon is not fully supported by EST, cDNA and RNA-seq data (Supplementary Figure S5). The real transcript appears to begin only after the annotated methionine, which would lead to translation starting in the second exon, giving rise to a full-length protein with all the canonical domains. Furthermore, this protein would present a molecular weight of 37 kD and a similar sequence to its paralog Pp3c17_20550V3.1.

After manually checking and curating the sequences, the remaining 16 SR proteins were aligned and compared with *A. thaliana* SR proteins. Importantly, all were unambiguously assigned to one of the six previously described subfamilies, despite the varying number of members within each subfamily. A protein domain search was then performed using MyHits^5^ to confirm that all *P. patens* SR proteins presented the characteristics of their assigned subfamily.

## Is the *Physcomitrella Patens* SR Protein Family Structurally Similar to That of Vascular Plants?

**FIGURE 2:**
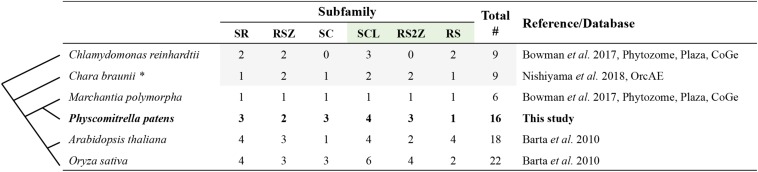
SR protein numbers in six representative plant species. The column on the right indicates the sources of the data. Gray shading indicates SR proteins requiring further annotation. For *Chara braunii*, marked with an asterisk, refer to Supplementary Table S1. Values in bold indicate *Physcomitrella patens* SR proteins manually verified and curated in this study. The green shaded subfamilies are plant-specific. Lines on the left are a schematic representation of the phylogenetic relationships among the species.

A comparison between the *A. thaliana* and *P. patens* SR protein families is shown in Figure 1, while Figure 2 and Supplementary Table S1 summarize the number of SR proteins within each subfamily among six representative species from different phylogenetic groups. The SR subfamily, comprised of four members in both Arabidopsis and rice, includes three proteins in *P. patens*, one in *Marchantia polymorpha* and two in both *C. reinhardtii* and *Chara braunii*. SR subfamily members all harbor two RRMs, sharing a conserved SWQDLKD motif in the second RRM as well as the characteristic RS region. All *P. patens* proteins from this subfamily contain a glycine-rich region separating the two RRMs, a trait shared by three SR subfamily Arabidopsis proteins but not present in At-SR30. The RSZ subfamily comprises three members in *A. thaliana* and *O. sativa*, only two in *P. patens*, *C. reinhardtii* and *C. braunii*, and one in *M. polymorpha*. It is characterized by the presence of a zinc knuckle between the RRM domain and the RS region, which is preceded by a glycine-rich region. Strikingly, the SC subfamily, while comprising only one member in *A. thaliana*, includes three in rice and four in *P. patens*, with all of these proteins containing one RRM followed by the RS region. *C. braunii* and *M. polymorpha* also have only one SC subfamily member, while the *C. reinhardtii* genome does not appear to code for any. The plant-specific SCL subfamily is present in all species analyzed, with *P. patens* and Arabidopsis possessing four members, rice six, and *C. reinhardtii, C. braunii* and *M. polymorpha* containing three, two and one, respectively. This subfamily is characterized by an N-terminal charged extension, in addition to the canonical RRM and RS region. The RS2Z subfamily is also plant-specific and is characterized by two zinc knuckles between the RRM and the RS region, as well as a C-terminal SP-rich region. Contrarily to the RSZ subfamily, there is no glycine-rich region after the RRM, with Arabidopsis and *C. braunii* containing two RS2Z members, rice four, *M. polymorpha* one, *P. patens* three and *C. renhardtii* none. Finally, the plant-specific RS subfamily includes four members in Arabidopsis, two in *O. sativa* and *C. reinhardtii*, but apparently only one in *C. braunii*, *M. polymorpha*, and *P. patens*. It is characterized by two RRMs, with the second containing no SWQDLKD motif, and an RS region. The different number of SR proteins within each subfamily among different plant species could have played a role in the adaptation to different habitats and perhaps even speciation, particularly if novel and distinct functions were acquired. Future comparative studies across species should reveal the functions of SR orthologs or, at the very least, provide insight into the evolutionary history of each lineage.

## Concluding Remarks and Outlook

Colonizing new habitats requires adaption to new environmental conditions, which can only be achieved by evolving traits that allow an organism to cope with such conditions. Given the documented role of alternative splicing in allowing plants to cope with adverse environments and its prevalence across the plant kingdom, it seems likely that this posttranscriptional mechanism was important in plant adaptation to terrestrial habitats. Naturally, it follows that if early land plants already modulated alternative splicing through SR proteins, then these RNA-binding factors could have provided an advantage in the adaptation to land. As mentioned above, comparative studies across species representative of the major plant groups should help understand how certain traits appeared and evolved. A number of comparative studies have already been conducted (Kalyna and Barta, 2004; Iida and Go, 2006; Kalyna et al., 2006; Richardson et al., 2011; Califice et al., 2012; Rauch et al., 2014), but as more genomes and transcriptomes become sequenced and annotations improve, more information can be extracted from these analyses, thus requiring continuous updates.

However, comparative studies require solid and reliable data that are easily accessible in public databases. In fact, an indispensable prerequisite to the comparison of the number of SR proteins across species is the availability of well-annotated genomes, transcriptomes, and proteomes. Recent years have seen substantial advances in this regard, but there are still numerous misannotations, including in protein sequences and domain composition, that significantly hamper gene classification and functional studies. In the case of our survey of SR proteins in the moss *P. patens*, we were able to unambiguously classify all SR genes into the previously established subfamilies, thus helping validate the definition proposed by Barta et al. (2010). Nevertheless, our analysis shows that care should be taken when working with the *P. patens* databases, as the genome is clearly not perfectly curated and annotated. A recent paper on *M. polymorpha* reported 17 *P. patens* SR proteins (Bowman et al., 2017), as they did not consider that Pp3c11_26740V3.1 and Pp3c11_26750V3.1 occupy the same position in the genome, likely because that information is not readily available in any database. Another study identifies 18 SR proteins in *P. patens*, though they do not discriminate between SR and SR-like proteins, thus overestimating the number of SR proteins (Fesenko et al., 2017). Furthermore, the authors place Pp3c7_5100V3.1 in the RS2Z subfamily despite the fact that it only contains one zinc knuckle, which underlines the confusion in nomenclature regarding this family. Earlier studies comparing SR proteins in plants (Califice et al., 2012; Rauch et al., 2014) and 27 eukaryotes (Richardson et al., 2011) report only 10 members of this gene family in *P. patens*, though also finding representatives for all the subfamilies. The tendency to report more SR genes in recent years reflects the improvement in the *P. patens* genome annotation. However, the gene models currently available in the several public databases, which are based on the current genome annotation, are not yet perfect, as in some cases they are not supported by expression data.

Regarding SR-like proteins, although they were formally excluded from the SR family when the nomenclature was updated a decade ago (Barta et al., 2010; Manley and Krainer, 2010), many studies still refer to them as such, as they also harbor both RRM and RS regions. *A. thaliana* expresses two SR-like proteins, but their number in *P. patens* remains uncertain. Our preliminary analysis identifies three (data not shown), but Fesenko and coworkers reported four (Fesenko et al., 2017). This discrepancy needs to be addressed in future studies.

Our analysis of the current *P. patens* genome annotation substantiates the existence of 16 canonical SR proteins distributed across the six previously described families. Conservation of the number of subfamilies and the protein structure organization of SR proteins from multicellular green algae to bryophytes to angiosperms, including the three plant-specific subfamilies, supports an ancient diversification of this family, likely predating the colonization of land habitats. Of note is the absence of the SC and the RS2Z subfamilies in *C. reinhardtii*. The SC subfamily, being also present in other eukaryotes including animals, could have been lost in this lineage. On the other hand, it is also possible that the plant-specific RS2Z subfamily had not evolved yet, appearing only in more complex algae, such as *C. braunii*. A number of evolutionary innovations previously assigned only to land plants have recently been identified in *C. braunii* (Nishiyama et al., 2018). It is tempting to speculate that the appearance of the RS2Z subfamily was one such innovation.

Experimental evidence for the conservation of function between *P. patens* and other plant model species is required to elucidate to which extent SR proteins already played a key regulatory role in both constitutive and alternative splicing when plants colonized terrestrial habitats. Additionally, a detailed functional analysis of *P. patens* SR proteins will help identify other mechanisms and signaling pathways in which these proteins could be involved. It is interesting to note that SR protein family composition varies greatly among plant lineages, both in total number and in subfamily size, suggesting a role for SR gene loss and gain in plant evolution and adaptation. Comprehensive comparative studies will ultimately shed new light on land plant evolution and elucidate the role of SR proteins in stress tolerance of early land plants.

## Author Contributions

JM, MK, and PD designed the study and wrote the manuscript. JM conducted the *in silico* analyses and prepared the figures.

## Conflict of Interest

The authors declare that the research was conducted in the absence of any commercial or financial relationships that could be construed as a potential conflict of interest.

## Footnotes

1 https://www.ncbi.nlm.nih.gov

2 https://genomevolution.org/coge/

3 https://bioinformatics.psb.ugent.be/plaza

4 https://phytozome.jgi.doe.gov/pz/portal.html

5 https://myhits.isb-sib.ch/cgi-bin/motif_scan
