# Supplementary material for: Current Challenges in Studying Alternative Splicing in Plants: The Case of Physcomitrella patens SR Proteins
Source: Front Plant Sci. 2020 Mar 24;11:286. doi: 10.3389/fpls.2020.00286 (PMC7105729; doi:10.3389/fpls.2020.00286)
Supplement: Supplementary file 1 [file Data_Sheet_1.PDF]

Pp3c11_26750V3.1 (Pp-RSZ25)

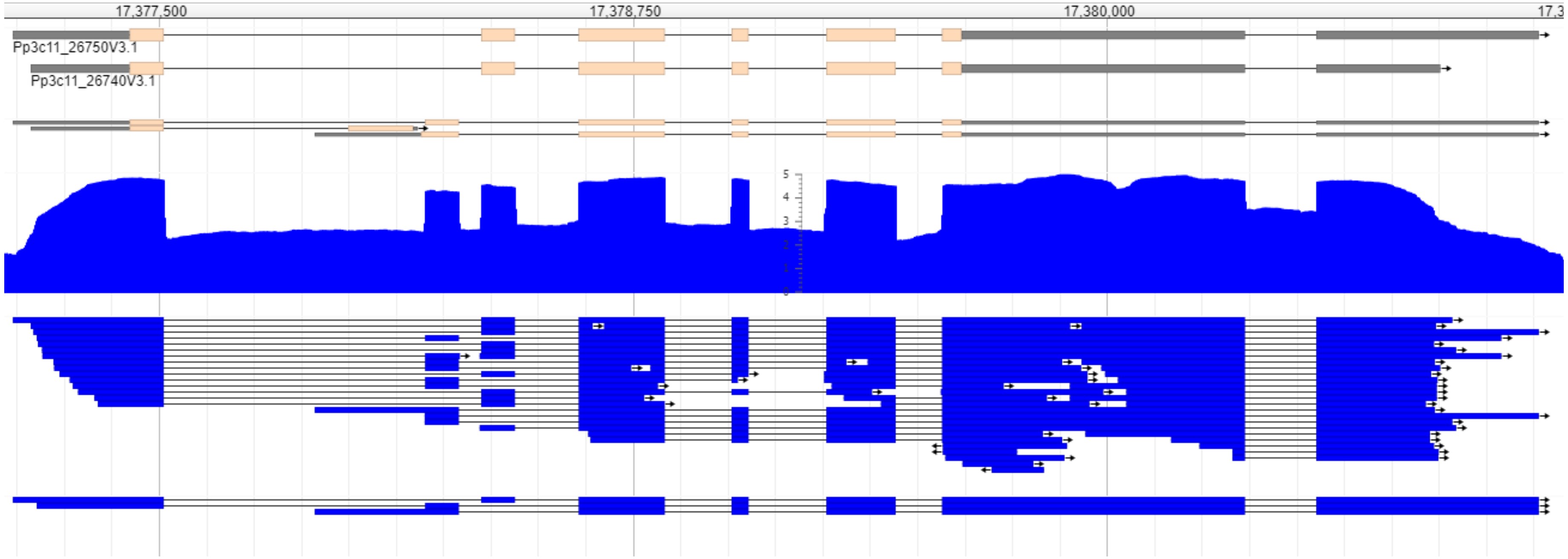

Figure S1. Printscreen from Phytozome JBrowse showing that Pp3c11_26740V3.1 and Pp3c11_26750V3.1 occupy the same position in the *Physcomitrella patens* genome.

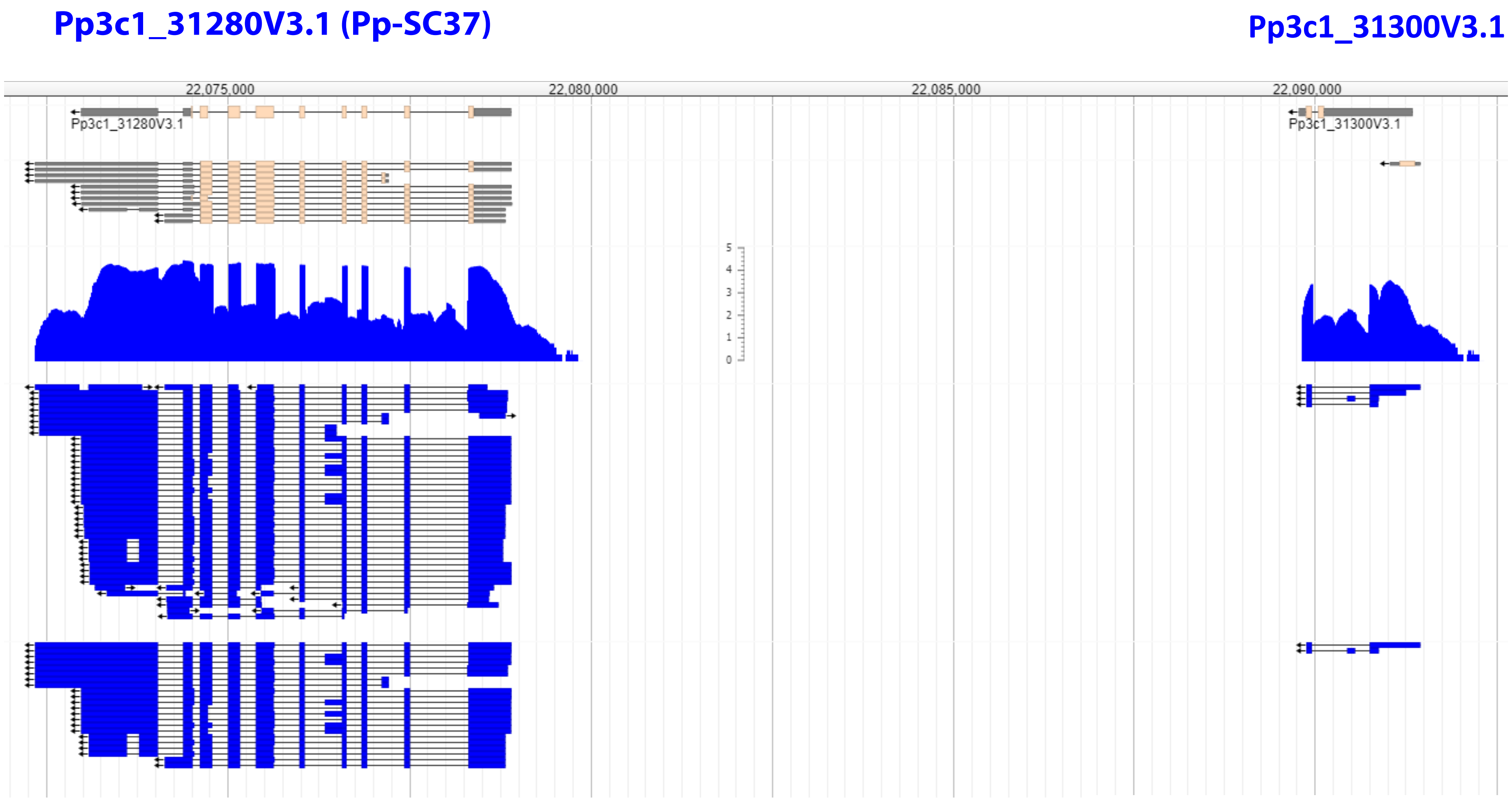

Figure S2. Printscreen from Phytozome JBrowse showing that candidate SR protein gene Pp3c1_31300V3.1, located next to Pp3c1_31280V3.1, is supported by EST, cDNA and RNA-seq data, but its sequence does not support a full-length protein.

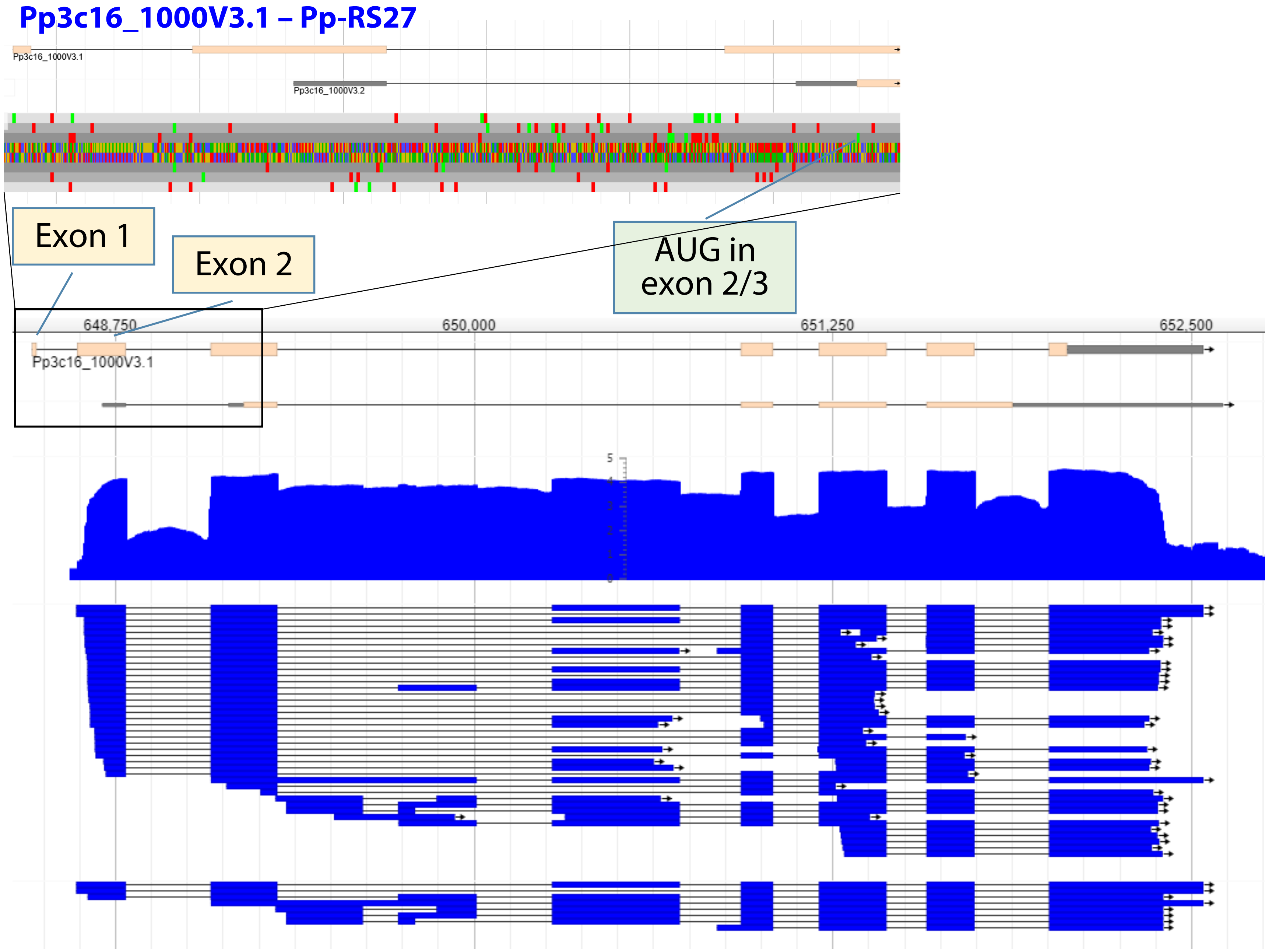

Figure S3. Printscreen from Phytozome JBrowse showing Pp3c16_1000V3.1, which does not have an annotated 5' UTR and whose first exon is not supported by EST, cDNA and RNA-seq data.

Pp3c20_7750V3.1 – Pp-RS2Z27

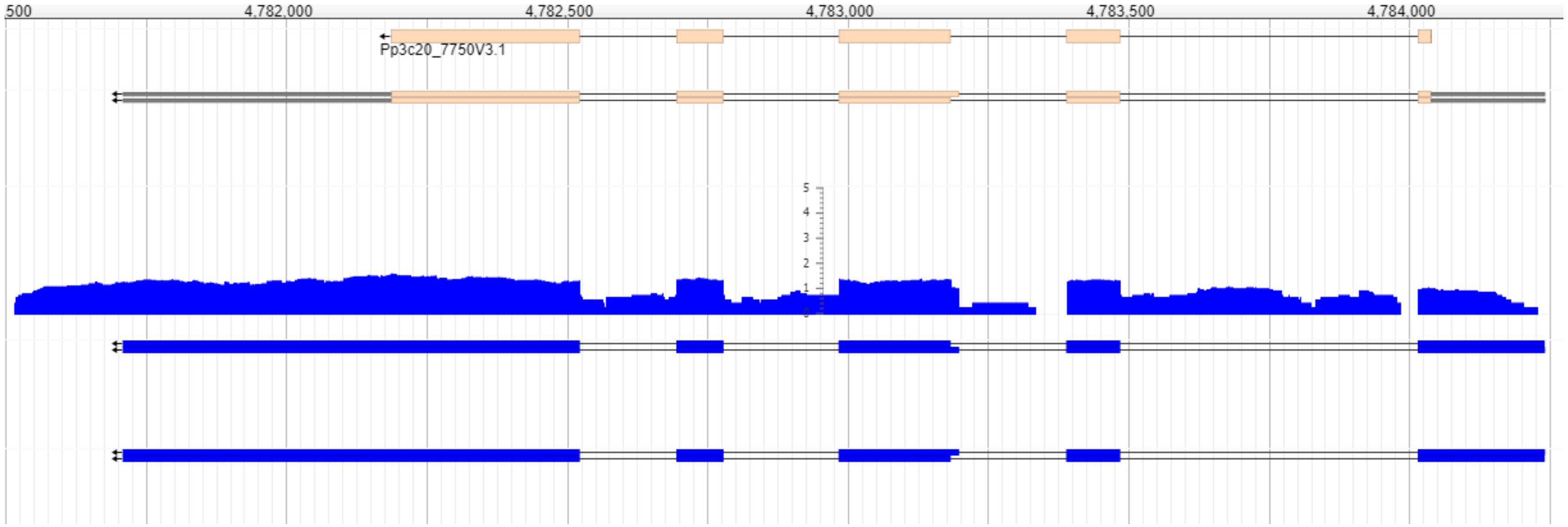

Figure S4. Printscreen from Phytozome JBrowse showing the lack of 5’ and 3’ UTRs in Pp3c20_7750V3.1, despite both being supported by EST, cDNA and RNA-seq data and being annotated in the alternative transcripts.

Pp3c14_23400V3.1 (Pp-RS2Z37)

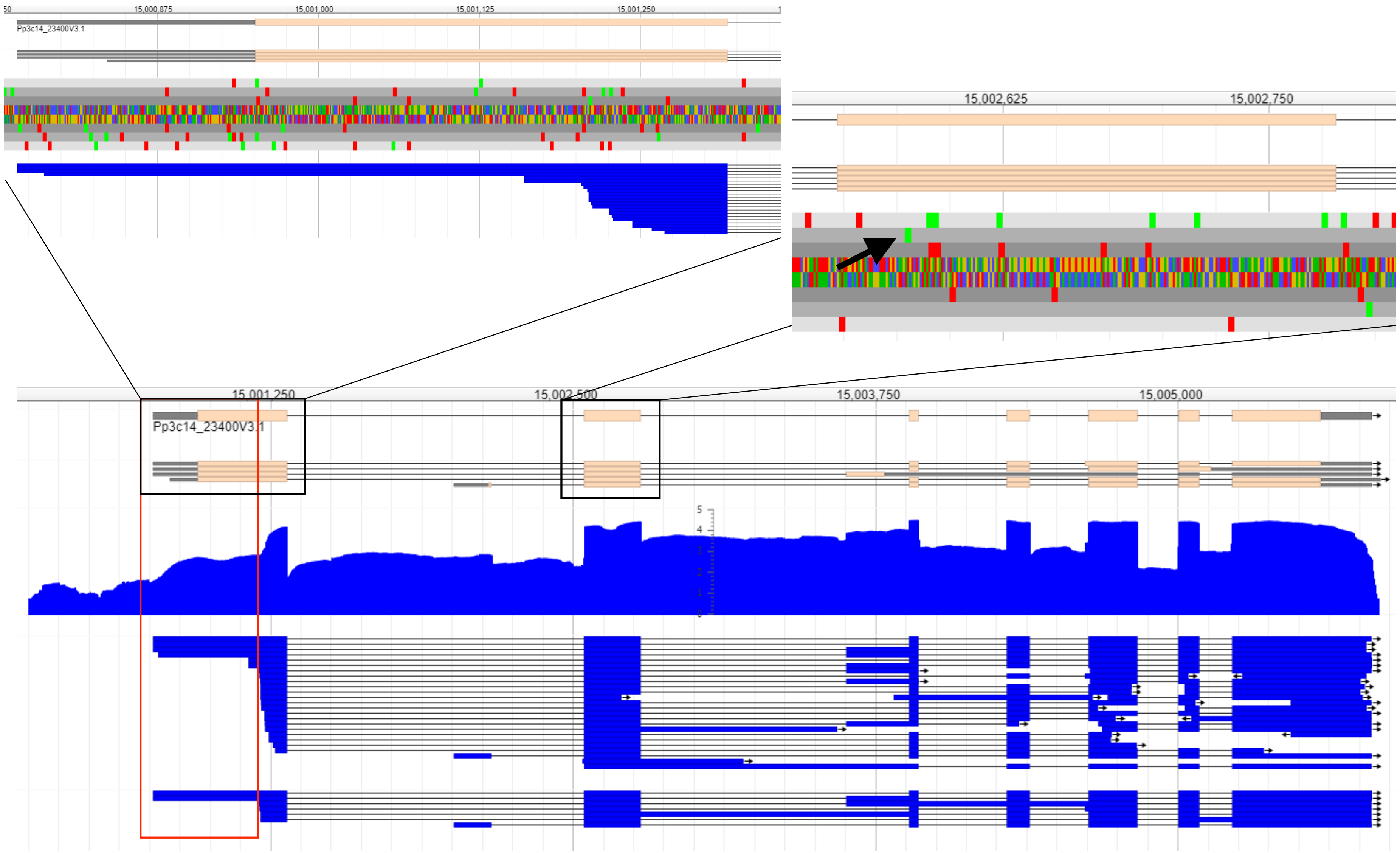

Figure S5. Printscreen from Phytozome JBrowse showing the case of Pp3c14_23400V3.1, whose first exon is not fully supported by EST, cDNA and RNA-seq data (red box), and the position of its first AUG supported by the experimental data (black arrow).

Table S1. SR proteins in *Chara braunii*

List of putative SR proteins in the algae *C. braunii* obtained by tBLASTn using the protein sequence of the 18 *Arabidopsis thaliana* SR proteins as queries. SR protein subfamilies shaded in green are plant-specific.

Name	Subfamily	Reported in Nishiyama et al. 2018	Our assessment	Notes
g12769	SR	Yes	Yes	Contains all the characteristics of an SR protein
g72806	SR	Yes	No	Short sequence containing just one RRM, identical to g12769. Appears to be a mis-annotation and not a real SR protein
g860	RSZ	Yes	Yes	Contains all the characteristics of an RSZ protein
g38426	RSZ	Yes	Yes	Contains all the characteristics of an RSZ protein
g34604	SC	Yes	Yes	Contains all the characteristics of an SC protein
g49492	SCL	Annotated as an RNA-binding protein	Yes	According to Nishiyama et al., 2018, it encodes an RNA-binding protein. It has all the characteristics of an SR protein
g34152	SCL	Annotated as having a Zn knuckle	Yes	According to Nishiyama et al., 2018, it encodes RS-containing Zinc knuckle protein. We did not find evidence of a Zn knuckle
g39174	RS2Z	Yes	Yes	Only has one Zn knuckle, but as it is a tandem duplicate of g39176. There is a high probability of also containing two ZnK
g39176	RS2Z	Yes	Yes	Contains all the characteristics of an RS2Z protein
g18868	RS	Yes	Yes	High homology to Arabidopsis RS subfamily, though the structure/sequence is probably mis-annotated, given the unusual length and partially missing genomic sequence
